# Hypophosphatemic rickets developed after treatment with etidronate disodium in a patient with generalized arterial calcification in infancy

**DOI:** 10.1016/j.bonr.2015.09.001

**Published:** 2015-09-09

**Authors:** Kentaro Miyai, Daisuke Ariyasu, Chikahiko Numakura, Kaori Yoneda, Hitoshi Nakazato, Yukihiro Hasegawa

**Affiliations:** a Division of Endocrinology and Metabolism, Tokyo Metropolitan Children's Medical Center, 2-8-29 Musashidai, Fuchu, Tokyo 183-8561, Japan; b Division of Developmental Genetics, Institute of Resource Development and Analysis, Kumamoto University, 2-2-1 Honjo, Chuo-ku, Kumamoto 860-0811, Japan; c Department of Pediatrics, Yamagata University School of Medicine, 2-2-2 Iida-nishi, Yamagata 990-9585, Japan; d Department of Pediatrics, Kumamoto University, 1-1-1 Honjo, Chuo-ku, Kumamoto 860-8556, Japan

**Keywords:** GACI, Generalized arterial calcification of infancy, ENPP1, ectonucleotide pyrophosphatase/phosphodiesterase 1, NPPH, nucleotide pyrophosphohydrolase, PPi, inorganic pyrophosphate, VSMCs, vascular smooth muscle cells, ARHR2, autosomal recessive hypophosphatemic rickets type 2, EHDP, etidronate disodium, Generalized arterial calcification of infancy, Hypophosphatemic rickets, Ectonucleotide pyrophosphatase/phosphodiesterase 1, Etidronate disodium

## Abstract

Ectonucleotide pyrophosphatase/phosphodiesterase 1 (*ENPP1*) was originally reported as a responsible gene for generalized arterial calcification in infancy (GACI). Though the prognosis of GACI patients is poor because of myocardial infarction and heart failure in relation to medial calcification of the coronary arteries, some patients rescued by bisphosphonate treatment have been reported. Recently, *ENPP1* is also reported as responsible for autosomal recessive hypophosphatemic rickets type 2. We show here a boy with homozygous *ENPP1* mutations diagnosed as having GACI in early infancy. After the diagnosis, he was treated with etidronate disodium (EHDP) in combination with antihypertensive drugs. The calcification of major arteries was diminished and disappeared by the age of eight months. He also showed mild hypophosphatemia (2.6–3.7 mg/dl) from the age of one year. After the treatment with EHDP for five years, he showed genu valgum with hypophosphatemia (2.6 mg/dl). He was diagnosed as having hypophosphatemic rickets at the age of seven years. The findings that hyper-mineralization of the arteries and hypo-mineralization of the bone observed in the same patient are noteworthy. *ENPP1* could be regarded as a controller of the calcification of the whole body at least in part.

## Highlights

• A boy with homozygous *ENPP1* mutation suffered GACI and subsequent hypophosphatemic rickets.
• *ENPP1* mutation caused both hyper-mineralization in the arteries and hypo-mineralization in the bone in the same patient.
• ENPP1 could be regarded as a mineralization controller of the body.

## 1 Introduction

Generalized arterial calcification of infancy (GACI; OMIM #208000) is a disorder characterized by medial calcification of elastic fibers in major arteries, such as aorta, renal arteries and coronary arteries, leading to angiostenosis throughout the body. Patients suffered from GACI show fetal distress, poor sucking and hypertension, and are often fatal within the first 6 months due to myocardial infarction and heart failure (Rutsch et al., 2001, Cheng et al., 2005).

*ENPP1* (ectonucleotide pyrophosphatase/phosphodiesterase 1) is reported as one of the responsible genes for GACI (Rutsch et al., 2001). ENPP1 has nucleotide pyrophosphohydrolase (NPPH) activity in the extracellular fluid generating inorganic pyrophosphate (PPi) and nucleotide monophosphate from nucleotide triphosphate. Accumulated PPi inhibits alkaline phosphatase (ALP) activity and mineralization through binding to hydroxyapatite crystals (Fleisch et al., 1966, Addison et al., 2007, Anderson et al., 2005). Therefore, loss-of-function mutations of *ENPP1* gene cause lack of PPi and up-regulating ALP activity, leading to promotion of mineralization in the vascular smooth muscle cells (VSMCs) (Villa-Bellosta et al., 2011, Zhu et al., 2011).

Recently, loss-of-function mutations of *ENPP1* gene were also found in patients with autosomal recessive hypophosphatemic rickets type 2 (ARHR2; OMIM #613312) by linkage analyses (Lorenz-Depiereux et al., 2010, Levy-Litan et al., 2010). To date, the mechanisms that loss-of-function mutations of *ENPP1* cause hyper-mineralization in the extra bone tissues and hypo-mineralization in the bone are still unclear.

Etidronate disodium (ethane 1-hydroxy-1, 1-diphosphonate; EHDP) is one of the first-generation bisphosphonates and its structure resembles that of pyrophosphate. It has been reported that treatment with EHDP improved the overall survival of patients with GACI by diminishing arterial calcification (Otero et al., 2013, Edouard et al., 2011, Galletti et al., 2011, Rutsch et al., 2008). Because the dose of EHDP required to inhibit bone resorption is near the one that impairs mineralization, EHDP could also serve as an inhibitor of mineralization in the bone and in the extra bone tissues (Fleisch, 2002).

We previously reported a boy who was diagnosed as having GACI with homozygous *ENPP1* gene mutations. He was treated with EHDP and antihypertensive drugs from the age of two months, and calcification of the arteries was disappeared by the age of eight months (Numakura et al., 2006). Afterwards he showed genu valgum with hypophosphatemia (2.7 – 3.7 mg/dl) at the age of five years and diagnosed as having hypophosphatemic rickets at the age of seven. Here we report his clinical course and discuss the role of ENPP1 in the mineralization in the bone and extra bone tissues.

## 2 Materials & methods

Written informed consent was obtained from the parents of our patient, and the study was approved by local ethical review board of our hospital.

### 2.1 Biochemical measurements

Serum calcium (Ca), phosphate (Pi), ALP levels were measured by standard colorimetric methods (SRL, Inc., Japan). Serum FGF23 level was measured by an ELISA kit (Kainos, Japan) which can only recognize the intact FGF23 (Yamazaki et al., 2002). Tubular reabsorption of phosphate (%TRP) was calculated by 100 × {1 − (urine Pi/serum Pi) / (urine Cr/serum Cr)}(%). Maximal tubular reabsorption of phosphate per GFR (TmP/GFR) was calculated by TRP × serum Pi.

## 3 Case report

**Fig. 1:**
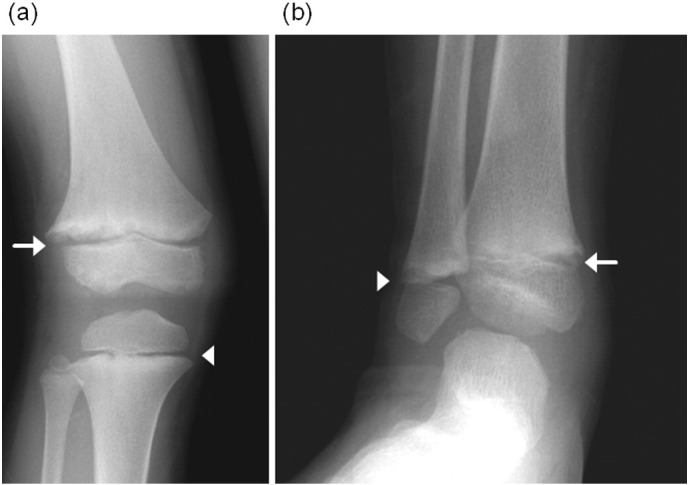
X-ray pictures of the knee (a) and the ankle (b) of the patient at the age of seven. (a) Metaphyseal fraying and flaring of the distal end of the femur (arrow) and metaphyseal fraying of proximal end of the tibia (arrowhead). (b) Metaphyseal fraying and flaring in the distal end of the tibia (arrow) and the fibula (arrowhead).

**Fig. 2:**
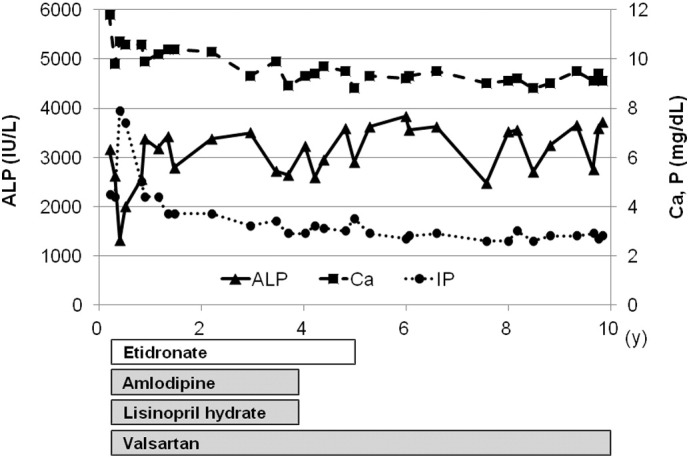
Biochemical parameters and the treatment regimen of the patient. After diagnosis, the patient was treated with EHDP and antihypertensive drugs. From infantile period, serum ALP remains high and serum phosphate remains low compared to age-related references.

A boy from the first-cousin parents was born by emergency cesarean section at 36 weeks gestation because of fetal distress. He showed systemic edema, hepatomegaly and hypertension up to 120 mmHg of systolic blood pressure. Calcification of the major arteries including aorta, carotid artery, renal artery and pulmonary artery was detected on whole body computed tomography (CT). He was diagnosed as having GACI. DNA analyses from the peripheral blood leukocytes showed that he had homozygous nonsense mutations of *ENPP1* gene (c.2188C > T, p.R730*) and his parents were heterozygous for the same mutation. NPPH activity of mutated ENPP1 was 4% compared to control (Numakura et al., 2006). A blood examination showed normal Ca (10.0 mg/dl), slightly decreased Pi (4.3 mg/dl), and high ALP levels (2683 IU/l) at the age of two months, when the treatment with EHDP at a dose of 18 mg/kg was started. To treat hypertension, antihypertensive drugs (amlodipine, lisinopril hydrate and varsartan) were also started, and then his systolic blood pressure was maintained below 100 mmHg. The calcification of the arteries was diminished and disappeared on CT scanning by the age of eight months. Because hypertension was improved, treatment with amlodipine and lisinopril hydrate was stopped at the age of four years. Treatment with EHDP was stopped by the age of five years, when he showed genu valgum of the both legs. At the age of seven years, X-ray of his knee and ankle showed flaying of metaphyseal bone (Fig. 1). A routine blood examination showed normocalcemia (8.8–10.4 mg/dl), hypophosphatemia (2.6–3.7 mg/dl), and high ALP (2591–3836 IU/l) continuously since he was at the age of one year (Fig. 2). Based on these examinations, he was diagnosed as having hypophosphatemic rickets. At the age of ten years, serum Pi, %TRP and TmP/GFR were 2.8 mg/dl, 90.3% and 2.5 mg/dl, respectively. No ectopic calcification was observed including major arteries, kidneys, joints, and spinal ligaments by the age of ten years, and his systolic blood pressure was maintained below 120 mmHg with varsartan. At the age of twelve years, high serum FGF23 level (84 pg/ml) was observed with low serum Pi level (2.8 mg/dl).

## 4 Discussion

We reported here a case with GACI subsequently developed hypophosphatemic rickets. To our knowledge, there are only three reports about GACI patients similar to our patient (Otero et al., 2013, Edouard et al., 2011, Rutsch et al., 2008).

One can easily question why GACI patients showed hypo-mineralization in the bone after hyper-mineralization in the extra bone tissues. The signs of GACI are usually evident in early infancy, even in prenatal period (Eronen et al., 2001, Crade et al., 1991, Nagar et al., 2003). On the other hand, hypophosphatemia or rickets in GACI patients were noted first at the age between two and three years (Edouard et al., 2011, Rutsch et al., 2008). To explain these phenotypic differences depending on age, we speculate possibilities of the change in the level of serum phosphate, production of FGF23 and treatment with EHDP.

Age dependent change in serum phosphate levels might explain the hyper-mineralization in the arteries observed in GACI patients. A previous report showed that high phosphorus diet exaggerated aortic calcification in *Enpp1* −/− mice by the age of two months. In addition, calcium content of aorta two months after transplantation from two-month-old wild type mice to *Enpp1* −/− littermates was significantly greater than that from wild type to wild type, but far lower than that from *Enpp1* −/− to *Enpp1* −/− (Lomashvili et al., 2014). These data suggest that high Pi/PPi ratio is responsible for arterial calcification in early infancy in mice. Circulating Pi is relatively higher in infancy than that in childhood and adulthood. Furthermore, serum phosphate level in cord blood was negatively related to gestational age (Fenton et al., 2011). Thus, high Pi/PPi ratio in early infancy and in fetal period might be critical for arterial mineralization. This speculation is supported by the fact that there are no reports about recurrence of arterial calcification after cessation of EHDP treatment in GACI patients except for one case whose arterial stenosis was worsened by calcitriol and phosphate supplementation (Otero et al., 2013, Rutsch et al., 2008, Numakura et al., 2006).

Hypophosphatemia and hypo-mineralization of the bone in GACI patients after infancy might be caused by FGF23 elevation resulted from progression of arterial calcification. VSMCs from *Enpp1 −*/*−* mice up-regulate the expression of *Fgf23* in a calcified environment (Tintut et al., 2003, Zhu et al., 2013), and those treated with recombinant Fgf23 reduced calcium deposition vice versa (Zhu et al., 2013). These data suggest that hypophosphatemia observed in patients with *ENPP1* mutation might be a byproduct of FGF23 up-regulation, in a hormonal manner, whereas VSMCs secret to protect themselves from progression of calcification in an autocrine or a paracrine manner. It is well known that FGF23 causes hypophosphatemia by inhibiting both activation of vitamin D and reabsorption of phosphate in the kidney. As shown in ARHR2 patients, *ENPP1* gene mutations are related to FGF23 elevation (Levy-Litan et al., 2010, Saito et al., 2011). In our patient, %TRP was relatively low under the condition of hypophosphatemia, and serum FGF23 level was high compared to those of normal subjects (10–50 pg/ml) (Yamazaki et al., 2002, Igaki et al., 2011). These biochemical data resembled the condition observed with X-linked hypophosphatemic rickets whose FGF23 levels are often elevated (Morey et al., 2011). It is still unclear whether the site of FGF23 production in ARHR2 patients and GACI patients is same.

Mineralization in the bone might be deteriorated by a long term EHDP treatment. Previous reports showed that patients who suffered such as Paget's disease and osteoporosis with long term EHDP treatment developed osteomalacia (Hoppé et al., 2012, Thomas et al., 1995, Siris et al., 1996). Moreover, a cessation of EHDP treated for a GACI patient led to improvement of rickets (Otero et al., 2013). It is known that the dose with which EHDP could inhibit bone resorption is near to that EHDP could inhibit mineralization by binding to the matrix vesicles necessary for precipitation and crystallization of calcium phosphate salts (Otero et al., 2013, Russell, 2011). Therefore, treatment with EHDP could inhibit calcification both in the arteries and in the bone.

It could be speculated that the arterial calcification is milder, which may not be clinically detected, in ARHR2 patients in infancy than that in GACI patients. The arterial calcification in ARHR2 patients disappears naturally with a decrease in serum phosphate level. These phenotypical variances might be explained by genotype of *ENPP1* mutation because a previous report showed that mild phenotype of GACI is in relation with a specific genotype, although there was a limitation with a small number of cohort (Rutsch et al., 2008).

**Fig. 3:**
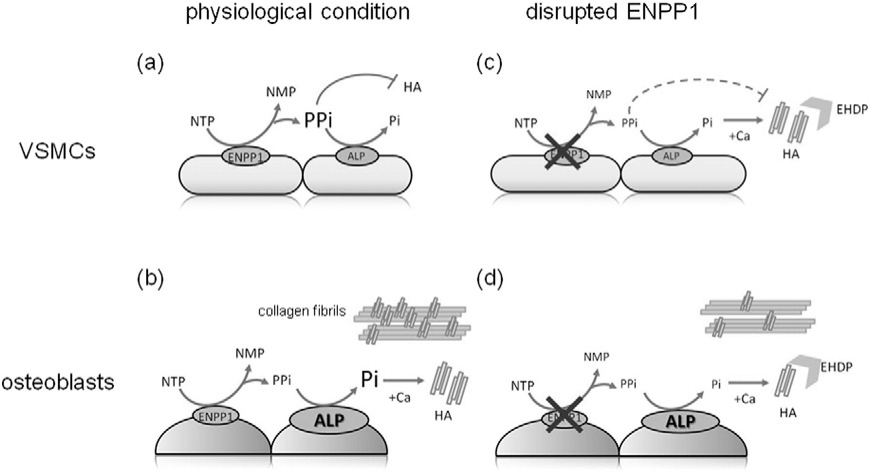
Schema of the calcification in the VSMCs and osteoblasts in a physiological condition and in a condition with disrupted ENPP1. In a physiological condition, accumulated PPi could inhibit hydroxyapatite (HA) crystallization in VSMCs (a), and inorganic phosphate could be generated from PPi by high ALP activity and used for the HA crystallization in the bone (b). In a condition of disrupted ENPP1, reduced production of PPi could lead to increased ALP activity and HA production would be increased in concert with circulating inorganic phosphate in VSMCs (c), and reduced inorganic phosphate generated form PPi could lead to reduced HA production which causes hypo-mineralization in the bone (d). EHDP could serve as inhibitor of HA crystals (c, d). NTP; nucleotide triphosphate, NMP; nucleotide monophosphate.

From these viewpoints, we speculated the role of ENPP1 in the mineralization. While ENPP1 is ubiquitously expressed in the body, activities of ALP are relatively high in osteoblasts compared to VSMCs (Gomez et al., 1995). When the expression of ENPP1 is disrupted, reduced extracellular PPi causes high ALP activities in both osteoblasts and VSMCs. Inorganic phosphate mainly supplied enterally could be used as a substrate of hydroxyapatite in the arteries as well as in the bone. Moreover, high inorganic phosphate in fetal, neonatal and infantile periods promotes calcification of VSMCs, and physiological decrease of inorganic phosphate by age might reduce the calcification in the arteries and in the bone. These temporal differences in the ratio of Pi to PPi may contribute to the difference of the mineralization in the body. Furthermore, EHDP could reduce hydroxyapatite leading to hypo-mineralization both in the arteries and in the bone (Fig. 3).

Clarifying the mechanisms of arterial calcification in GACI patients would lead to prevention of arterial calcification in CKD patients. Medial calcification of arteries is also related to patients with chronic kidney disease (CKD) (Chowdhury et al., 2004). Pyrophosphate could inhibit vascular calcification despite elevated calcium and phosphate concentration in vitro (Lomashvili et al., 2004), and treatment with pyrophosphate inhibits arterial calcification in uremic rats (O'Neill et al., 2011). These results support that Pi/PPi ratio is the important regulator of arterial mineralization in uremic patients as is predicted in patients with GACI.

In conclusion, we reported here a patient with homozygous *ENPP1* mutation who suffered hypophosphatemic rickets subsequent to GACI. Clarifying the mechanisms of mineralization defect of these patients could further lead to better understanding of the physiological control of mineralization by ENPP1 in the bone and the extra bone tissues.

## Disclosure statement

The authors have nothing to disclose.
